# *MLL5* improves ATRA driven differentiation and promotes xenotransplant engraftment in acute promyelocytic leukemia model

**DOI:** 10.1038/s41419-021-03604-z

**Published:** 2021-04-06

**Authors:** Diego A. Pereira-Martins, Isabel Weinhäuser, Juan Luiz Coelho-Silva, Pedro L. França-Neto, Luciana Y. Almeida, Thiago M. Bianco, Cleide L. Silva, Rafael F. França, Fabiola Traina, Eduardo M. Rego, Jan Jacob Schuringa, Antonio R. Lucena-Araujo

**Affiliations:** 1 Department of Hematology, Cancer Research Centre Groningen, University Medical Centre Groningen, University of Groningen, Groningen, The Netherlands; 2 Center for Cell-Based Therapy, University of Sao Paulo, Ribeirao Preto Sao Paulo, Brazil; 3 Hematology Division, LIM31, Faculdade de Medicina, University of Sao Paulo, Sao Paulo, Brazil; 4 Department of Genetics, Federal University of Pernambuco, Recife, Brazil; 5 Department of Medical Images, Hematology, and Clinical Oncology, University of Sao Paulo at Ribeirao Preto Medical School, Ribeirao Preto Sao Paulo, Brazil; 6 Department of Virology, Aggeu Magalhaes Institute/Oswaldo Cruz Foundation, Recife, Brazil

**Keywords:** Cancer models, Acute myeloid leukaemia, Mechanisms of disease, Stem-cell differentiation, Prognostic markers

## Abstract

Although the *mixed lineage leukemia 5* (*MLL5*) gene has prognostic implications in acute promyelocyte leukemia (APL), the underlying mechanism remains to be elucidated. Here, we demonstrate the critical role exerted by *MLL5* in APL regarding cell proliferation and resistance to drug-induced apoptosis, through mtROS regulation. Additionally, *MLL5* overexpression increased the responsiveness of APL leukemic cells to *all-trans* retinoic acid (ATRA)-induced differentiation, via regulation of the epigenetic modifiers *SETD7* and *LSD1*. In silico analysis indicated that APL blasts with MLL5^high^ transcript levels were associated with retinoic acid binding and downstream signaling, while MLL5^low^ blasts displayed decreased expression of epigenetic modifiers (such as *KMT2C*, *PHF8* and *ARID4A*). Finally, APL xenograft transplants demonstrated improved engraftment of *MLL5*-expressing cells and increased myeloid differentiation over time. Concordantly, evaluation of engrafted blasts revealed increased responsiveness of MLL5-expressing cells to ATRA-induced granulocytic differentiation. Together, we describe the epigenetic changes triggered by the interaction of MLL5 and ATRA resulting in enhanced granulocytic differentiation.

## Introduction

The *mixed lineage leukemia 5* (*MLL5*) gene, also known as the *lysine N-methyltransferase 2* (*KMT2E*), was originally proposed to be a functional member of the MLL family of proteins due to domain similarities^1^, despite of functional^2,3^ and structural^4^ disparities. Nevertheless, MLL5 interacts with the chromatin via its plant homeodomain (PHD) zinc finger to bind to the trimethylation of lysine 4 of histone 3 (H3K4me3) via a noncanonical binding mechanism^5^. In the hematopoietic tissue, MLL5 has been implicated to be involved in terminal myeloid differentiation, hematopoietic stem cell self-renewal^6^, and retinoic acid-induced granulopoiesis of normal human promyelocytes^7^. Yet, little is known about its pathophysiological importance in hematological malignancies.

In the clinical setting, previous studies have demonstrated that altered *MLL5* transcript levels have prognostic implications in acute myeloid leukemia (AML)^8^ and acute promyelocytic leukemia (APL)^9^. In AML, the functional effect of *MLL5* overexpression was only described regarding increased therapeutic response to decitabine, a DNA hypomethylating agent^10^, resulting in terminal differentiation. In the context of APL, the effects of MLL5 regarding the differentiation therapeutic agent (*all-trans* retinoic acid, ATRA), and its effect on disease burden and therapy response remains unclear. Here, we find that *MLL5* overexpression led to enhanced APL response to *all-trans* retinoic acid (ATRA) and promoted primary APL engraftment with increased granulocytic differentiation in a xenograft model.

## Material and methods

### Lentiviral vectors and lentivirus production

Recombinant lentivirus encoding the PML-RARA fusion transcript (bcr1 isoform) and the Hs*MLL5α* gene (hereinafter called *MLL5* gene) were generated using pCDH1-MCS1-EF1-GFP-Puro (pMEG) lentivector (#CD713B-1; System Biosciences, USA) in HEK293T cells according to the three-plasmid packaging procedure as described elsewhere^11^. The complete optimized sequence of *MLL5* gene was synthesized and fully sequenced in both directions by GenScript (Piscataway Township, USA). Lentiviral particles to overexpress *MLL5* gene were used to infect primary human leukemic blasts, NB4 and NB4-R2 cell lines. Particles containing the PML-RARA gene were used to transduce the U937 cells. Cells were sorted based on their GFP protein expression, and posteriorly used for in vitro assays. The efficiency of infection was further confirmed by gene expression quantification and western blotting analysis (Supplementary Fig. 1). For MLL5 knockdown, two sequences for shMLL5 (shMLL5#1 - TRCN0000154711 and shMLL5#2 - TRCN0000358557; Sigma-Aldrich) vectors were properly expanded and validated as previously described^12^. A shRNA sequence that does not target human genes (referred to as scrambled) was used as a control (hereinafter called shCTRL).

### Cell lines and drugs

All cell cultures were maintained in a humidified atmosphere at 37 °C with 5% CO_2_. NB4 (all-trans retinoic acid, ATRA-sensitive) and NB4-R2 (ATRA-resistant) cell lines were kindly provided by Dr. Pier Paolo Pandolfi (Harvard Medical School, USA), and maintained in RPMI 1640 (Gibco, USA) supplemented with 10% fetal bovine serum (FBS) (Gibco, USA), l-glutamine (2 mM), 1% penicillin/streptomycin (Invitrogen, USA). Mycoplasma contamination was routinely tested. All leukemia cell lines were authenticated by short tandem repeat analysis. The HEK293T (CRL-3216), U937 (CRL 1593-2™) and HS27A (CRL-2496) cell lines were obtained from the American Type Culture Collection and grown in DMEM (for HEK293T and HS27A; Gibco, USA) or RPMI (for U937; Gibco, USA) with 10% FBS. All-trans retinoic acid (ATRA) and arsenic trioxide (ATO) were obtained from Sigma-Aldrich (St. Louis, USA) and cytarabine (citarax) was obtained from Blau pharmaceuticals (Sao Paulo, Brazil).

### Patient samples

Bone marrow samples of APL patients were studied after informed consent and protocol approval by the Ethical Committee in accordance with the Declaration of Helsinki (registry #12920; process number #13496/2005; CAAE: 155.0.004.000-05). Mononuclear cells (MNCs) were isolated via Ficoll (Sigma-Aldrich) separation and cryopreserved.

### Gene set enrichment analysis (GSEA) for MLL5 biological pathways in APL

Gene set enrichment analysis (GSEA) was performed using the Broad Institute software (http://software.broadinstitute.org/gsea/index.jsp). All genes from the RNA-seq of the TCGA AML cohort were pre-ranked according to their differential expression (fold change) and APL patients (*n* = 16) were categorized into high and low expression of *MLL5*, using their median expression rate as a cut-off. Enrichment scores (ES) were obtained with the Kolmogorov-Smirnov statistic, tested for significance using 1000 permutations, and normalized (NES) to consider the size of each gene set. As suggested by the GSEA, a false discovery rate (FDR) cut-off of 25% (FDR *q*-value <0.25) was used^13^. The limma-voom tool (http://usegalaxy.org) was used to examine differentially expressed genes and genes with ≥1 log difference and adjusted *p*-value of <0.05 were considered significant^14^. Data visualization was performed with the ClustVis platform^15^.

### In vitro primary APL co-cultures and ex vivo cultures

HS27A cells were plated on gelatine coated culture flasks and expanded to form a confluent layer. Next, APL cells were cultured in Gartner’s medium consisting of α-MEM (Thermo Scientific) supplemented with 12.5% fetal bovine serum (Gibco), 12.5% horse serum (Gibco), 1% penicillin and streptomycin, 2 mM glutamine (Gibco), 57.2 mM β-mercaptoethanol (Merck Sharp & Dohme BV), 1 mM hydrocortisone (Sigma-Aldrich) and 20 ng/mL G-SCF, TPO and IL-3. Co-cultures were grown at 37 °C and 5% CO2 and demi-populated weekly for counting. To evaluate the cell proliferation, the GFP content was measured at day 0 in 1 × 10^5^ primary APL transduced cells (transduction efficiency) and co-cultured with HS27A cells for 14 days. The percentage of GFP positive (GFP^+^) cells was subsequently analyzed at day 7 and day 14. An increase in GFP^+^ cells was interpretated as cell proliferation of the transduced cells. Granulocytic differentiation was evaluated in primary co-cultures after 8 days of ATRA treatment (1 µM). At the end, cells were analyzed by FACS and histological evaluation was performed by May-Grünwald-Giemsa (MGG) staining. Images of MGG stained cells were made with a DM3000 (Leica) microscope.

### Western blot analysis

Equal amounts of protein were used as total extracts, followed by SDS-PAGE, Western blot analysis with the indicated antibodies. For imaging the SuperSignal^TM^ West Dura Extended Duration Substrate System (Thermo Fisher Scientific, USA) and Gel Doc XR + system (Bio-Rad, Hercules, CA, USA) were used. Antibodies against CDKN1A (sc-71811), MLL5 (sc-377182), OP18 (sc-55531), and α-tubulin (sc-5286) were obtained from Santa Cruz Biotechnology (San Jose, CA). Antibodies against H3K4me3 (#9727) and fibrillarin (#2639) were obtained from Cell Signaling Technology (Danvers, USA). All membranes were incubated with a primary antibody following manufacturer’s instructions (see Supplementary Table S1). Band intensities of cropped gels, which retained important bands were measured by the UN-SCAN-IT gel 6.1 software (Silk Scientific; USA).

### Quantitative real-time PCR

Total RNA from U937 (transduced with control and PML-RARA), NB4 and NB4-R2 at basal conditions (transfected with control [empty vector and scrambled] or transduced with the *MLL5* vectors [overexpression and silence]) or treated with ATRA (1 µM) at different time-points was obtained using the Trizol reagent (Thermo Fisher Scientific, USA). The cDNA was synthesized from 1 µg of RNA using High-Capacity cDNA Reverse Transcription Kit (Thermo Fisher Scientific). Following total RNA extraction, real-time quantitative polymerase chain reaction (RQ-PCR) assays with sample-derived cDNA were performed in duplicate on MicroAmp optical 96-well plates using a 7500 Real-Time PCR System (Applied BioSystems, USA) with the ACTB and GAPDH Standard Kit (#4310881E) as endogenous control. The gene expression of *MLL5*, *SETD7* and *SETD9* genes was determined using TaqMan Gene Expression Assay (*MLL5*: Hs00218773_m1, *SETD7*: Hs00363902_m1 and *SETD9*: Hs04187070_m1; Applied BioSystems), following the manufacturer’s instructions. For *LSD1, PHF8* and *RARB* quantification, the RQ-PCR was performed using Power SYBR Green Master Mix (Thermo Fisher Scientific) with specific primers for isoform A (referred *LSD1A*, forward: GTGGTAACAGGTCTTGGAGGG; reverse: CGTTGGCTTCATAAAGTGGGC), isoform b (referred *LSD1B*, forward: GACCGCCCTATGCAAGGAAT; reverse: GGGGGATTCGCTTCCAACTC), *PHF8* (forward: GGGAAGAACCAACAACGCAG; reverse: TCTGGACGATAGCGCGG), *RARB* (forward: TCGGCACACTGCTCAATCAAT; reverse: TACACTCGAGGGGGAGGAAG), *ACTB* (Beta-actin, forward: AGGCCAACCGCAAGAAG; reverse: ACAGCCTGGATAGCAACGTACA) and *GAPDH* (*GAPDH*, forward: GGACTCATGACCACAGTCCAT; reverse: GCCATCACGCCACAGTTT) as endogenous control. The gene expression of the target genes was calculated relative to a reference cDNA (wild type NB4 cell line) and set to 1. In all experiments, the same reference cDNA was used as an internal control to ensure that the results would be fully comparable among experiments. The gene expression values of the genes of interest were calculated as relative quantification using the ∆Ct method and expressing the results as 2-^ΔΔCt^, in which ΔΔCt = ΔCt_sample_ – ΔCt_NB4 or NB4-R2 cell line_. All the results regarding the gene expression analysis, were normalized as a fold relative to wild-type NB4 cells. As a comparative control, we included the *MLL5* gene expression evaluated in our own cohort of APL patients^9^, which was also normalized to the NB4 wild-type control (Supplemental Fig. 1C, D).

### Microarray and RNA-seq data analysis

Microarray and RNA-seq data were obtained from the following datasets: GSE19201^16^, GSE18397^17^, and GSE12662^18^, which were deposited in the GEO database (https://www.ncbi.nlm.nih.gov/geo). For the first two datasets, we investigated the mRNA expression of *MLL5*, *LSD1*, *SETD7*, and *SETD9* genes in samples from wild type NB4 cells, treated with vehicle (DMSO) and ATRA (1 µM) at different time-points (24, 48 and 72 h). For the last dataset, we investigated the expression of the *MLL5* gene in U937 cells transduced with an empty vector and Zinc-inducible promoter for the *PML-RARα* gene, after 6 and 9 h of Zinc treatment. Genes that presented normalized counts equal to 0 for any replicates were excluded from the analysis.

### Chromatin immunoprecipitation analysis sequence (ChIP-seq)

ChIP-seq data were obtained from the GSE18886 dataset^19^, which was deposited in the GEO database (https://www.ncbi.nlm.nih.gov/geo). APL cell models (NB4 cells, primary APL blasts or PML-RARA transduced U937 cells) were treated 24 h with ATRA (1 µM) and ChIP-seq was performed according to the previously described.

### In vitro assays

#### Cell proliferation

Cells were treated with thymidine (2 mM; CallBiochen, USA) for 18 h twice to induce cell cycle arrest at the G1/S boundary. Cells were subsequently seeded in 6-well plates and 1 million cells were collected and fixed with 70% ethanol at distinct timepoints: Day 1, 2, 3, 4, 6 and 8 and stored at −20 °C. Ki-67 staining was performed following the manufacturer’s instructions (Ki-67 PE clone SolA15; BioLegend, USA). Next, the mean of fluorescence intensity (MFI) was obtained by flow cytometry standard techniques using a FACSCalibur instrument (Becton-Dickinson, USA). IgG isotype was used as a negative control for each condition. As a confirmatory assay, we performed an MTT assay to measure cell viability. Transduced APL cell lines (2 × 10^4^ cells/well) were cultured in a 96-well plate in RPMI medium containing 10% FBS, during 1, 2, 4, 6 and 8 days. At the end of each culture period, 10 µL of a 5 mg/ml solution of MTT was added to each well followed by an incubation of 4 h at 37 °C. The reaction was stopped using 100 µL of 0.1 N HCl in anhydrous isopropanol. Cell viability was evaluated by measuring the absorbance at 570 nm, using the iMark™ Microplate Absorbance Reader (Bio-Rad, Richmond, CA, USA). In parallel, cells were seeded at a density of 1 × 10^4^ cells/ml in 10-cm dishes and the cell number was counted daily for seven days.

#### Cell cycle analysis

**Fig. 1:**
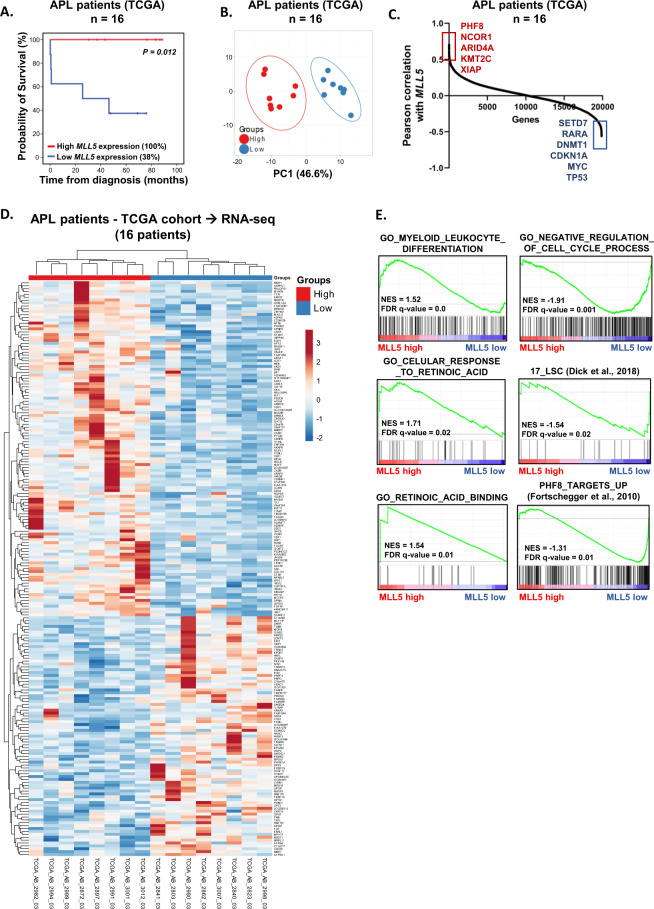
Differential expression of MLL5 defines different biology in APL patients. The probability of overall survival in patients with APL included in the TCGA study according to *MLL5* gene expression (**A**). Survival curves were estimated using Kaplan–Meier method, and the log-rank test was used for comparison. **B** Principal component analysis showing the differences in gene expression for APL patients included in the TCGA cohort dichotomized by *MLL5* expression (high and low expression). Pearson correlation shows the positively and negatively correlated genes with *MLL5* gene in APL patients (TCGA cohort). **C** Heatmap of differentially expressed genes (supervised clustering) in APL samples from TCGA cohort dichotomized according *MLL5* gene expression (dichotomization point: median value; groups: high and low expression; *n* = 8 in each group). **D** Gene set enrichment analysis (GSEA, lower panels) on a ranked gene list based on the leading-edge genes for *MLL5* expression in 16 de novo APL patient samples from TCGA study. Genes were ranked based on Pearson correlation with *MLL5* gene expression. Normalized enrichment score (NES) and false discovery rate (FDR) was used for significance.

Cell cycle phases were determined by BD Cycletest^TM^ Plus DNA Reagent Kit (Becton-Dickinson) according to the manufacturer’s instructions. A total of 4 × 10^5^ synchronized cells at G1/S phase were seeded in 24-well plates and collected and fixed at distinct timepoints: 0, 6, 8, 10 and 12 h. DNA content distribution was acquired with the FACSCalibur cytometer (Becton-Dickinson) and analyzed with the FlowJo software (Treestar, Inc., USA). Data from 12 h was used to generate the plots in Fig. 1C.

#### Apoptosis assay

For the apoptosis analysis, 5 × 10^5^ cells were seeded in 24-well plates and incubated in complete medium for 24 h in the presence of vehicle, ATO (1 µM, alone or in combination with 1 µM of ATRA) and cytarabine (Ara-C, 10 nM). The apoptosis rate was determined using the Annexin V-APC and propidium iodide (PI) binding assay (BD Biosciences, San Jose, CA, USA). All specimens were acquired by flow cytometry (FACSCanto; Becton-Dickison) and analyzed with the FlowJo software (Treestar, Inc., USA). All experiments were performed in triplicate and for each sample a minimum of 10 000 events were acquired.

#### Assessment of mitochondrial reactive oxygen species (mtROS) production

For this purpose, 1 × 10^6^ cells were treated with ATO (1 µM) for 12 h. Following the manufacturer’s instructions mtROS levels were determined with the fluorogenic MitoSOX RedTM (Thermo Fisher Scientific) via flow cytometry procedures (FACSCalibur, Becton-Dickinson) and analyzed with the FlowJo software (Treestar, Inc.). Hydrogen peroxide (0.1 µM) was used as a positive control for the experiment.

#### Granulocytic differentiation induction

2 × 10^5^ transduced NB4 and NB4-R2 cells were cultured in RPMI medium containing 10% FBS in the presence of ATRA (1 µM) alone or in combination with ATO (1 µM) for 48 and 72 h at 37 °C (DMSO and NaOH were used as vehicle control for ATRA and ATO, respectively). The differentiation rate was determined by evaluating the number of CD11b-, CD11c-, CD15-, and CD16-positive cells (percentage and MFI levels). Experiments were conducted using the FACS LSRII flow cytometer (Becton-Dickinson) and analyzed with the FlowJo software (Treestar, Inc.). In parallel, cytospin preparations stained with May-Grünwald-Giemsa (MGG) were used to evaluate morphological changes in NB4 cells treated with ATRA (1 µM) for 5 days (compared to DMSO control).

#### Colony formation assay

Colony formation capacity was evaluated out in semisolid methylcellulose medium (1.5 × 10^3^ cells/ml; MethoCult 4230; StemCell Technologies Inc., Canada). Clonogenic assays were performed in the presence of medium alone (without serum), vehicle (DMSO control 0.01%) and ATRA (0.1 and 0.5 µM). Colonies were detected after 10 days of culture by adding 1 mg/ml of MTT reagent and scored with the Image J quantification software (US National Institutes of Health, USA).

### In vivo APL xenotransplant

All animals were housed under specific pathogen free conditions in individually ventilated cages during the whole experiment. The animals were maintained according to the Guide for Care and Use of Laboratory Animals of the National Research Council, USA, and to the National Council of Animal Experiment Control recommendations.

Eight week old female NSGS (NOD.Cg-Prkdcscid Il2rgtm1Wjl Tg(CMV-IL3,CSF2,KITLG)1Eav/MloySzJ) mice were purchased from the Jackson Laboratory. Mouse experiments were performed in accordance with the national and institutional guidelines, and all experiments were approved by the Institutional Animal Care and Use Committee of the University of Sao Paulo (IACUC-USP; Process number #067/2018). Mononuclear cells were isolated from six different APL patients (each one isolated from a different APL patient; clinical characteristics in Supplemental Table S2) were depleted for CD3^+^ cells and transduced twice with empty vector or MLL5 vector (multiplicity of infection, MOI > 50) using Retronectin-coated plates (Takara). Forty-eight hours post transduction, GFP levels were checked by FACS (empty vector (mean ± Standard Deviation): 20.5 ± 2.5% and MLL5: 4.8 ± 1.9%) and 1 × 10^6^ transduced cells were directly injected into the tibia of the animals (*n* = 6 for each group). Human CD45^+^ levels were measured regularly in blood obtained by sub-mandibular bleeding and mice were sacrificed after engraftment was confirmed (on a set time point of 12 weeks). Cells from the mouse organs including BM, spleen and liver were isolated and analyzed for the presence of human CD45^+^ cells, regardless of GFP expression (to analyze the transduced and non-transduced human cells). Using FACS analysis, we evaluated the presence of human APL blasts and the more differentiated myeloid committed cells defined by the expression of CD45^+^CD117^+^CD33^+^HLADR^−^CD11b^−^CD19^−^ and CD45^+^CD117^−^CD33^+^CD11b^+^ markers respectively^20^, inside the GFP^−^ (non-transduced) and GFP^+^ (transduced cells) cell populations. All antibodies used for the staining were incubated following manufacturer’s instructions (see Supplementary Table S1). Left over cells from BM were sorted for human APL blasts: GFP^+^CD45^+^CD117^+^CD33^+^ cells, to perform ex vivo cultures and stored in liquid nitrogen.

### Statistical analysis

According to survival receiver operating characteristic (ROC) curve analysis, the median value of *MLL5* expression was used to dichotomize patients into two groups (i.e., low expression, <4910; high expression, ≥4910). Overall survival was defined as the time from diagnosis to death from any cause; those alive or lost to follow-up were censored at the date last known alive. For patients who achieved CR, DFS was defined as the time from CR achievement to the first adverse event: relapse, development of secondary malignancy, or death from any cause, whichever occurred first. All P values were two sided with a significance level of 0.05. All statistical analyses were performed using the statistical package for the social sciences (SPSS) 19.0 and R 3.3.2 (The CRAN project, www.r-project.org) software.

## Results

### *MLL5* expression correlates with enhanced retinoic acid response in APL patients

Increased *MLL5* expression was reported to be associated with improved prognosis in APL when treated with ATRA plus anthracycline-based chemotherapy^9^. To validate our findings, we analyzed the *MLL5* gene expression in APL patients, using the TCGA public dataset^21^. Our analysis indicated increased overall survival in APL patients with high *MLL5* expression (Fig. 1A). Principle component analysis using the transcriptome of APL patients (included in the TCGA cohort) demonstrated distinguished gene expression signature between the two groups (high *MLL5* expression vs Low *MLL5* expression) (Fig. 1B). Interestingly, APL patients with high *MLL5* expression were associated with increased expression of epigenetic modifiers (*KMT2C*, *PHF8*, and *ARID4A*) (Fig. 1C, D). Subsequent, gene set enrichment analysis (GSEA) associated MLL5^high^ APL patients with the terms “Retinoic acid binding”, “Myeloid leukocyte differentiation” and “Cellular response to retinoic acid”, while low *MLL5* APL patients were associated with “negative regulation of cell cycle”, “17-leukemic stem cell markers” and “genes up-regulated upon PHF8 knockdown” (Fig. 1E). As a next step, we decided to evaluate the function of high and low MLL5 expression in vitro using APL cell lines.

### MLL5 significantly impacts on APL cell proliferation and cell cycle progression

The human coding sequence of the *MLL5* gene was stably overexpressed in NB4 and NB4-R2 cell lines. To confirm the overexpression of MLL5 at the protein level, protein lysates from transduced cell lines were prepared according to the described methods. As shown in Supplemental Fig. 1A, B, cells infected with pMEG-MLL5 and shMLL5 lentivirus efficiently overexpressed and downregulated the MLL5 protein (in comparison with the Empty vector and shScrambled, respectively). The level of *MLL5* expression post transduction was compared to the level of *MLL5* detected in APL patients in a previous study^9^. As depicted by supplemental Fig. 1C, D, the overexpression and knockdown of *MLL5* in APL cell lines corresponded to patients with high and low *MLL5* expression, respectively.

**Fig. 2:**
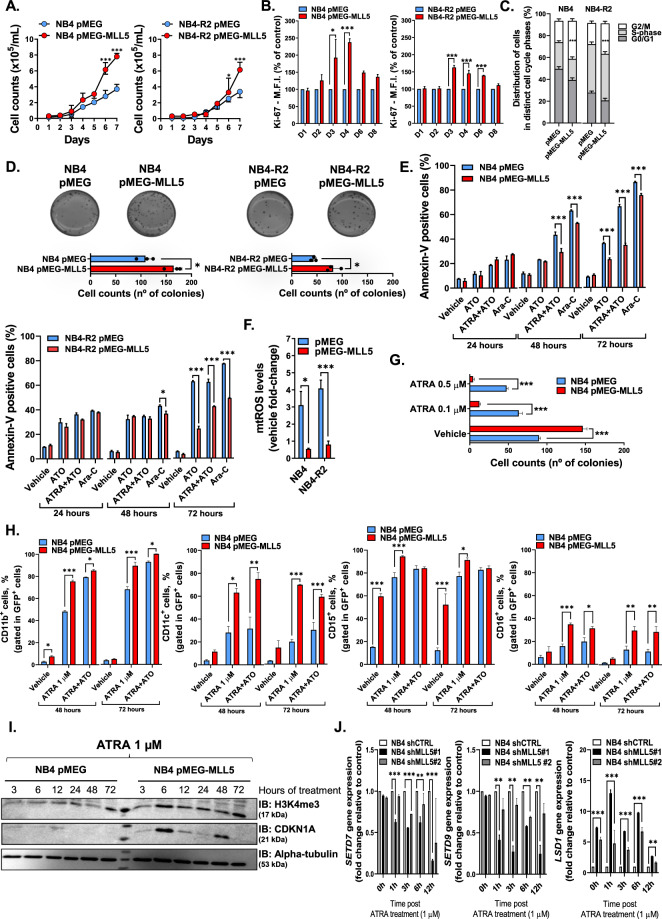
Effect of MLL5 on proliferation assays. **A** Growth curves, **B** Ki67 staining and **C** cell cycle analysis of NB4 and NB4-R2 cell lines lentivirally transduced with *MLL5* or empty vector (pMEG, control). **D** Representative example of one of four independent experiments of colony formation assay in methylcellulose using lentivirally transduced NB4 and NB4-R2 cell lines. Graphic bars represent the number of colony-forming cells in each well. **E** Graphic bars represent the number of colony-forming cells in each well in NB4 cells transduced with MLL5 or empty vector (pMEG, control) treated with vehicle (DMSO, control) and ATRA (0.1 and 0.5 µM). Effect of MLL5 on drug-induced apoptosis assay. **F** Percentage of apoptotic cells after 24, 48 and 72 h in culture with apoptotic stimulus (ATRA 1 µM; ATO 1 µM and AraC 10 nM). **G** Mitochondrial reactive oxygen species (mtROS, MitoSOX™ 5 µM) measured by FACS in lentivirally transduced cell lines after 12 h of treatment with ATO (1 µM). All experiments were performed in triplicate. Effect of *MLL5* on myeloid differentiation. **H** Percentage of CD11b^+^, CD11c^+^, CD15^+^ and CD16^+^ in NB4 cells infected with empty vector or pMEG-*MLL5* lentiviruses after 48 (left panel) and 72 (right panel) h of ATRA (1 μM) alone or in combination with ATO (1 µM each) treatment used as the standard stimulus for differentiation. Effect of *MLL5* knockdown on myeloid differentiation. **I** Western blot for H3K4me3, CDKN1A (p21) of total protein lysates from NB4 cell line infected with empty vector or pMEG-*MLL5* lentiviruses and treated with ATRA 1 µM. A total of 30 µg of protein was loaded in each lane. **J** Gene expression analysis of *SETD7*, *SETD9* and *LSD1* in NB4 cell line infected with shRNAs targeting *MLL5* (*shMLL5*) and scrambled controls (shCTRL) upon ATRA treatment (1 µM). The expression of *SETD7*, *SETD9* and *LSD1* genes was quantified by Real-time quantitative PCR (RQ-PCR) using *GAPDH* and *ACTB* as endogenous control. Note: Data from continuous variable were all expressed as mean ± standard error of the mean. *P < 0.05. ***P < 0.001. NS indicates not significant.

Daily cell count of NB4 and NB4-R2 cell lines overexpressing MLL5 indicated increased cell proliferation (Fig. 2A), which was further confirmed by an MTT assay (Supplemental Fig. 2A) and Ki-67 staining (Fig. 2B). Furthermore, MLL5 overexpression resulted in accumulation of cells in the G2/M phase and enhanced colony formation capacity (Fig. 2C, D). Conversely, these effects were abrogated upon short hairpin knockdown of *MLL5* (Supplemental Fig. 2B–E). In summary our data suggests that MLL5 promotes APL cell proliferation and colony formation by accelerating cell cycle progression.

### MLL5 overexpression results in increased APL cell viability and resistance to drug induced apoptosis through mtROS regulation

Next, we treated the transduced APL cell lines with arsenic trioxide (ATO), ATO + ATRA and cytarabine (Ara-C) for 24, 48 and 72 h. Overexpression (Fig. 2E) and knockdown (Supplemental Fig. 3A, B) of MLL5 led to increased resistance to drug-induced apoptosis in a time-dependent manner.

Studies reported that the encoded MLL5 protein modulates intracellular reactive oxygen species (ROS) levels^22^. Given, that ATO induces ROS accumulation^23^ we hypothesized that MLL5 overexpression affects ATO induced mitochondrial ROS (mtROS) generation. Our analysis indicated a 3-fold decrease of mtROS levels in *MLL5*-transduced and ATO stimulated cells compared to control (Fig. 2F), but no difference in sh*MLL5* cells (Supplemental Fig. 3C). Altogether, these findings demonstrate that MLL5 overexpression endows APL cells with a growth advantage and enhanced ROS clearance, which culminates in increased resistance to arsenic/anthracycline drug-induced apoptosis.

### MLL5 sensitizes APL cells to ATRA-induced granulocytic differentiation via the recruitment of an epigenetic machinery

Earlier publications revealed that MLL5 is implicated in terminal myeloid and retinoic acid-induced differentiation^6^. Given that ATRA is the standard therapeutic agent in APL, we decided to investigate how MLL5 affects ATRA-induced myeloid differentiation in APL, using the ATRA responsive NB4 cell line. Interestingly, while the overexpression of MLL5 drives proliferation and clonogenicity (Fig. 2A–D), we noticed a significant reduction of colonies in MLL5 overexpressing cells upon ATRA stimulation (Fig. 2G). To evaluate differentiation, we analyzed the expression of the granulocytic/monocytic differentiation markers CD11b, CD11c, CD15 and CD16 in NB4-MLL5 cells at basal conditions and when treated with ATRA (alone or in combination with ATO, 1 µM) (Fig. 2H, Supplemental Fig. 4A). Intriguingly, CD11b and CD15 expression was increased in NB4-MLL5 cells at basal conditions, while the treatment with ATRA ± ATO further enhanced the expression of all differentiation markers compared to control cells. In accordance with our FACS data, morphological cell evaluation revealed that NB4-MLL5 cells treated with ATRA (1 µM, 5 days) exhibited a decreased nucleus-cytoplasm ratio with nuclear lobulation, suggestive of neutrophilic differentiation, which was not observed in control cells (Supplemental Fig. 4B). Moreover, also MLL5 transduced NB4-R2 cells presented increased expression of CD15 at basal conditions. Overall, NB4-R2-MLL5 cells displayed enhanced granulocytic differentiation after 48 h and 72 h of ATRA + ATO treatment (Supplemental Fig. 4C). These results suggest that the ATRA and ATO-mediated disruption of the PML-RARA fusion protein in APL cells plays an essential role in MLL5-induced granulocytic differentiation.

**Fig. 3:**
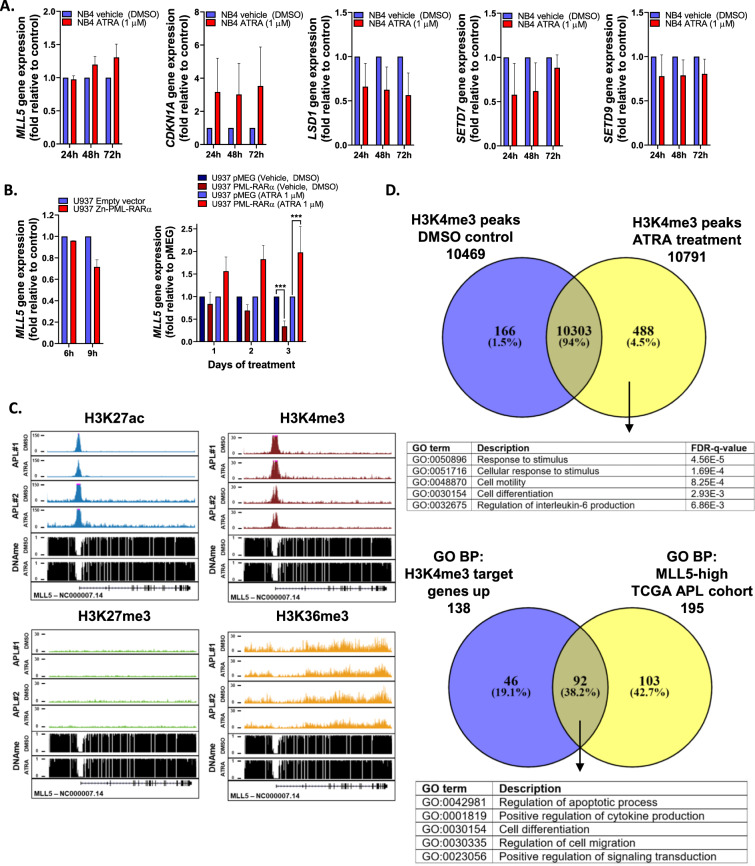
Molecular effects of ATRA treatment in PML-RARα+ cells. **A** Gene expression analysis of *MLL5*, *LSD1*, *SETD7*, *SETD9*, and *CDKN1A* in NB4 cell line treated with vehicle (DMSO) and ATRA (1 µM) for 24, 48 and 72 h. Expression data was retrieved from GSE19201 and GSE18397. **B** Gene expression analysis of *MLL5* in U937 cell line infected with empty-vector and Zinc-inducible PML-RARα (left panel). The expression of *MLL5* gene was quantified 6 and 9 h after Zinc administration. Gene expression analysis of *MLL5* in U937 cell line infected with empty-vector and PML-RARα (bcr1 isoform; right panel). Cells were treated with ATRA (1 µM) and DMSO control for 1, 2 and 3 days. Data were expressed as mean ± standard error of the mean. **C** Overview of APL ChIP-seq (H3K27ac, H3K4me3, H3K27me3 and H3K36me3) data at the genomic regions of the MLL5 gene before and after ex vivo ATRA treatment. **D** Upper panel exhibit the overlap in H3K4me3-bound loci determined by ChIP seq in APL patient#1. Peaks were distinguished in present only in DMSO control, only upon ATRA treatment and in both conditions. Overall, 488 genes were enriched only upon ATRA treatment, which were associated with 138 gene ontology (GO)—biological process (BP). Lower panel exhibit the GO BP for H3K4me3 up regulated genes present only upon ATRA-treatment and the GO BP found in APL patients from the TCGA cohort with high MLL5 expression. ChIP-seq data was retrieved from GSE18886. *P < 0.05. **P < 0.01. ***P < 0.001. NS indicates not significant.

Since PML-RARA recruits a repressive epigenetic complex, which dissociates upon ATRA treatment via PHF8 activation (causing H3K4 hypermethylation)^24^, we investigated the effect of MLL5 on trimethylation of H3K4 in response to ATRA. After 6 h of ATRA treatment (1 µM), we observed a significant increase of the H3K4me3 and CDKN1A (Fig. 2I) in MLL5-expressing cells followed by an up-regulation of the histone-modifying enzyme *SETD7* gene after 12 h of treatment (Supplemental 4D). *PHF8* expression was significantly increased in NB4-MLL5 cells treated with ATRA alone after 72 h, while these effects were observed already at 24 h when ATRA was combined with ATO (Supplemental Fig. 4E). As a result of this activation, the *RARB* expression (the main downstream target of PHF8, which promotes promyelocytic differentiation) was significantly up-regulated in MLL5-overexpressing cells upon ATRA and ATRA + ATO treatments (Supplemental Fig. 4F). Contrarily, shMLL5 cells exhibited a consistent down-regulation of the *SETD7* and *SETD9* genes, while the *LSD1* gene was up-regulated (Fig. 2J). To validate our findings, we evaluated the expression of *MLL5* and its partners using non-transduced NB4 cells treated with ATRA (1 µM) at different time-points. As expected, we detected an up-regulation of the *CDKN1A* and *MLL5* genes after 48 and 72 h of treatment (Fig. 3A). Additionally, upon ATRA treatment, NB4 cells presented a time-dependent down-regulation of the *LSD1* gene expression but no difference for *SETD7* and *SETD9* genes (Fig. 3A). Using a zinc-inducible model for PML-RARA expression in U937 cells, we observed a reduction of *MLL5* levels over time when PML-RARA expression was induced. These effects were abrogated by the administration of ATRA, which restored the transcript levels of *MLL5* (Fig. 3B).

Next, we examined the MLL5 promoter region, to evaluate the main epigenetic marks in APL patients treated with ATRA and control (DMSO)^25^. We noticed that upon ATRA treatment, no changes were observed for H3K27me3 and H3K36me3 tracks, while only minor changes were observed for H3K4me3 and H3K27ac at the *MLL5* locus (Fig. 3C). Subsequently, we evaluated the gene ontology (GO, biological process (BP)) process associated with the genes that had significant chromatin occupancy for H3K4me3 upon ATRA-treatment (since high MLL5 expression increased H3K4me3 in our in vitro model). A total of 488 genes were only present in the ATRA group (Fig. 3D, upper panel), which were associated with 138 GO BP processes (Fig. 3D). Interestingly, comparing the GO BP signature using the APL patients included in the TCGA cohort with high *MLL5* expression, we noticed a high overlap between the GO process (Fig. 3D, lower panel). Gene ontology analysis for terms present in both MLL5high APL patients and H3K4me3 ATRA only were associated with “Positive regulation of cytokine production”, “cell differentiation” and “regulation of apoptotic process”, suggesting that APL patients with high *MLL5* expression presented an intrinsic biology more compatible with ATRA-induced changes on H3K4me3 (Supplementary Table 3).

### Ex vivo evaluation of primary APL blast cells expressing MLL5 present similar phenotypes to APL cell lines

**Fig. 4:**
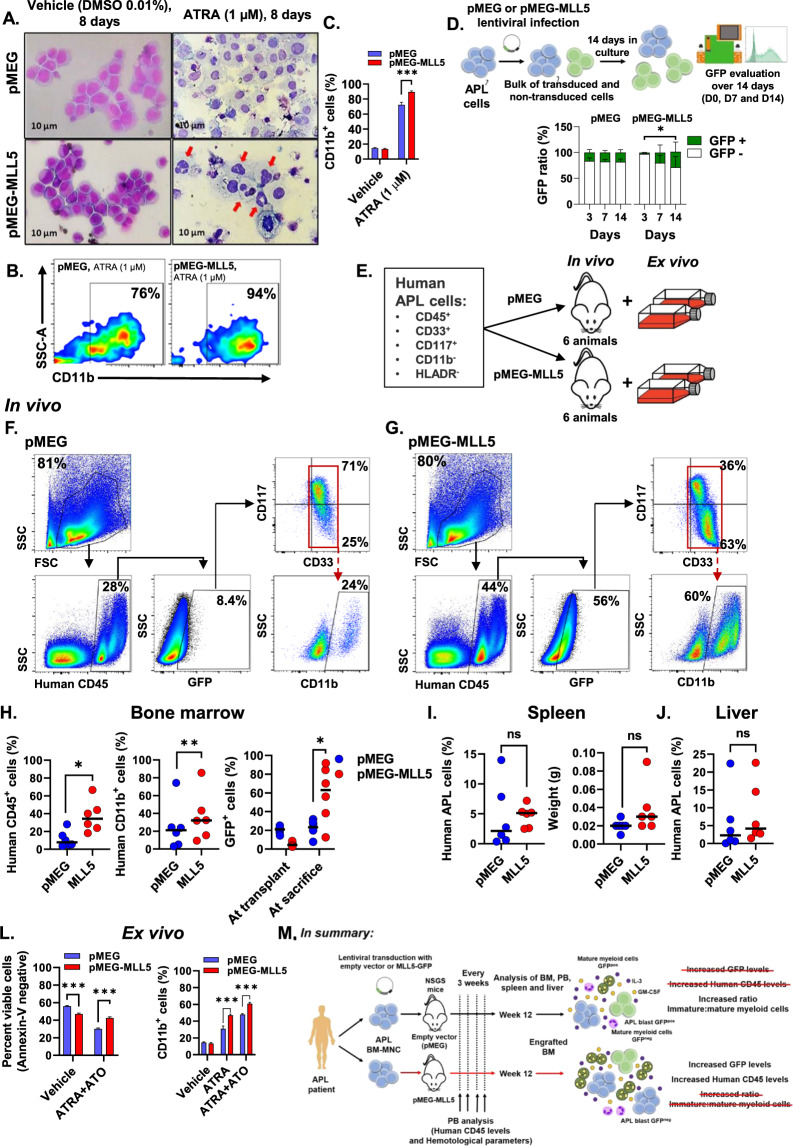
Primary MLL5 transduced APL blasts (GFP^+^ cells) exhibited increased cell proliferation and granulocytic differentiation in vitro and in vivo. **A** Representative images of May-Grünwald-Giemsa-stained cytospins of transduced primary APL blasts (pMEG, and pMEG-MLL5) treated 8 days with ATRA (1 µM) and DMSO control (vehicle, 0.01%). **B** Fluorescence-activated cell sorting (FACS) immunophenotype of CD11b staining of transduced APL blasts with *MLL5* or empty vector and **C** percentage of CD11b^+^ cells in transduced primary blasts after 8 days of ATRA (1 μM) treatment as the standard stimulus for differentiation. Data were expressed as mean ± standard error of the mean. **D** Schematic representation of cell proliferation assay based on GFP expression in primary APL cells transduced with MLL5 and the empty vector control (pMEG). Graphic bars represent the number of GFP^+^ cells inside the bulk of transduced primary APL blasts during 14 days of culture. Data were expressed as mean ± standard error of the mean. Overview of the mouse xenograft for APL. **E** Schematic representation of the generation of the xenograft mouse model for APL engraftment using NSGS mice. Representative FACS phenotype from a primary murine bone marrow transplanted with human transduced APL blasts with the empty vector (**F**) or the *MLL5* gene (**G**) at sacrifice. APL blasts and mature myeloid committed cells were analyzed by flow cytometry using markers against CD117, CD33 and CD11b as indicated (inside the population huCD45^+^ and GFP^+^). Scatter plots showing engraftment of donor human CD45^+^ cells (regardless the GFP expression), human CD45^+^GFP^+^ cells and human CD45^+^GFP^+^CD11b^+^ cells in bone marrow (**H**), **I** spleen (percentual of engraftment and spleen weight) and **J** liver of transplanted mice at sacrifice. Data were expressed as median values. Ex vivo analysis of transduced APL blasts reinforces in vitro findings. **L** Incubation of bone marrow sorted APL blasts cells (GFP^+^CD45^+^CD117^+^CD33^+^) from pMEG/MLL5 engrafted mice, with ATRA plus ATO (1 μM each) led to increased induction of apoptosis over the course of 72 h in empty vector cells. Percentage of CD11b^+^ cells in sorted blasts from murine BM isolated from engrafted pMEG and MLL5 mice, after 8 days of ATRA alone or in combination with ATO (1 μM each) treatment as the standard stimulus for differentiation. Data were expressed as mean ± standard error of the mean. **M** Summary results from APL xenograft murine model to study the role of MLL5 in APL. *P < 0.05. **P < 0.01. ***P < 0.001. NS indicates not significant.

Since APL cell lines (NB4 and NB4-R2 cells) exhibit aberrant karyotypes that are not commonly found in APL patients^26^, we decided to study the role of *MLL5* expression in primary APL blasts. Concordant with our cell line data, we observed increased differentiation upon ATRA treatment assessed by CD11b expression and cellular morphology (Fig. 4A–C). Morphological analysis of empty-vector (pMEG) and MLL5 transduced primary blasts treated with DMSO presented features of immature APL blasts, while ATRA treatment induced increased differentiation in MLL5-transduced cells (red arrows, Fig. 4A) compared to control. Additionally, we observed ex vivo clonal expansion of GFP^+^ MLL5-transduced cells over time, while no difference was observed for the empty vector controls (Fig. 4D). Similar to our findings in APL cell lines, the MLL5 overexpression in primary APL blasts enhanced cell proliferation and potentiated the effect of ATRA induced differentiation.

### MLL5-expressing APL blasts promote increased engraftment in a xenograft model, associated with increased granulocytic differentiation

Finally, we generated a xenograft mouse model using primary APL samples transduced with the pMEG-MLL5 or controls to assess the impact of MLL5 on APL cell viability and differentiation in vivo (Fig. 4E). At week 12, the engraftment of human APL cells was confirmed in both groups and the animals were sacrificed to analyze the level of human APL blast infiltration (transduced and non-transduced pMEG and pMEG-MLL5 cells) in bone marrow (BM), spleen, and liver. Interestingly, the BM of MLL5-transplanted mice presented increased levels of human CD45 + cells, GFP expression and frequency of CD11b+ compared to control (Fig. 4F–H). This result suggests that MLL5 promoted expansion of the transduced cells (GFP^+^) and enhanced myeloid differentiation, which was not observed in the GFP^-^ population (Supplementary Fig. 5A). Furthermore, MLL5-transduced cells showed inferior engraftment compared to controls (Fig. 4H), reinforcing the idea that MLL5 induces APL cell proliferation (Supplementary Fig. 5C, first panel). No differences in engraftment/weight were observed in the liver and spleen between MLL5 and control mice (Fig. 4I, J). Weekly hematological counts revealed no differences in peripheral blood leukocyte and platelet counts, while we observed a reduction in hemoglobin levels in empty vector mice at later time points (week 12) (Supplementary Fig. 5B).

To further study cellular characteristics of engrafted MLL5 cells, BM cells from empty vector and *MLL5*-transplanted mice were sorted (based on GFP^+^ and human CD45^+^CD33^+^CD117^+^ expression) (supplementary Table 2 shows the *MLL5* gene expression in the murine BM of sacrificed animals) for ex vivo experiments. Sorted GFP^+^ human APL blasts were cultured for 8 days with ATRA (1 µM) and ATRA + ATO (1 µM each) to determine induction of granulocytic differentiation or ATRA + ATO to evaluate drug induced apoptosis (since ATRA alone induces very low levels of apoptosis^27^). Xenograft derived ex vivo cultures, revealed that *MLL5*-expressing cells presented decreased sensitivity to ATO induced apoptosis after 72 h of treatment and higher CD11b levels at day 8 of ATRA administration compared to control cells (Fig. 4L). Interestingly, the number of MLL5 transduced apoptotic cells was slightly increased (inferior to 10%) when treated with the vehicle compared to pMEG cells. This could be attributed to the enhanced proliferative rate of MLL5 cells, which leads to a quicker exhaustion of nutrients. These results are in accordance with the previous in vitro and patient ex vivo observations, showing, that MLL5 enhances ATRA-induced differentiation in APL, which could be confirmed in a xenograft mouse model (Fig. 4M).

## Discussion

Here, we demonstrated for the first time that increased MLL5 expression improves ATRA-driven therapeutic response in APL. Since, high *MLL5* transcript levels were associated with favorable prognosis in APL patients treated with ATRA and anthracycline-based chemotherapy^9^, we hypothesized that this could emanate from a lower tumor burden or higher therapy response. However, when *MLL5* was overexpressed in APL cells we observed increased proliferation and resistance to drug induced apoptosis. Contrarily, in the presence of ATRA the proliferation rate of MLL5 was severely impaired and concomitantly the granulocytic differentiation rate was enhanced. These effects were mediated via a sustained increase of H3K4me3 across the genome and the modulation of histone-modifying enzyme-related genes. Interestingly, the modulation of histone-modifying enzymes was only observed in the context of MLL5 knockdown, but not when MLL5 was overexpressed. Moreover, the extent of the biological effect observed upon MLL5 silencing was correlated to the efficiency of the respective short hairpin to downregulate MLL5 expression. One reason why we could only observe a change in histone-modifying enzyme expression when MLL5 was silenced, could be because of the changes in interactomes when MLL5 is overexpressed^10^. To our knowledge, this is the first study to evaluate the interplay between MLL5 and SETD7/9 and LSD1, using both overexpression and knockdown models in APL. In line with previous publications, Sebastian et all (2009)^3^, demonstrated the indirect effect of MLL5 on H3K4 trimethylation via the regulation of SETD7/9 and LSD1 in myoblasts, in the context of MLL5 knockdown cells. Similar results were reported by our group in AML cell lines in vitro and in vivo (Almeida et al, 2018). While our study appears to be in contradiction with previous reports at first, it is critical to mention that the function of MLL5 in AML was investigated in the context of epigenetic-targeted therapy. Hence, our and previous studies reinforce the idea that the function of MLL5 can be orchestrated by epigenetic changes and that the classification of MLL5 as a pro/anti-tumorigenic gene should be considered within context.

In addition, the reduction of MLL5 expression was previously associated with changes at the H3K4 but not H3K9 histone mark^17,21^. Despite the up regulation of H3K4me3 observed in vitro, APL patients presented only a modest increase for H3K4me3 and H3K27ac at the MLL5 locus upon ATRA treatment^17,21^. We also noticed that the biological process observed in APL patients with high MLL5 levels overlapped with genes harboring high H3K4me3 chromatin-occupancy upon ATRA treatment. These observations suggest that patients with high MLL5 levels exhibit similar genetic signatures as APL samples treated with ATRA. Considering cell cycle progression, Deng et al. reported that ectopic overexpression of *MLL5* leads to cell cycle arrest in G1-phase^28^, while Cheng et al. observed cell cycle inhibition upon *MLL5* knockdown^29^. It is not uncommon that overexpression or knockdown of the same gene, causes analogous biological effects, indicating tissue dependent functions for *MLL5*. Future studies should consider the use of inducible models for MLL5 knockdown/overexpression to provide better comparability among the different phenotypes associated with MLL5 modulation in leukemic cells.

Furthermore, we observed increased resistance of MLL5 cells upon ATO and Ara-C treatment. Studies suggested that the loss of MLL5 leads to extensive DNA damage and elevated ROS levels^22^. Considering that ATO-mediated toxicity partly relies on ROS production to induce PML nuclear body formation and p53 pathway activation^23^, it is probable that the resistance of MLL5 overexpressing cells is linked to reduced mtROS generation.

Although, APL xenotransplants are extremely challenging, we managed to provide a model, which satisfactory recreates the disease to investigate APL genetic modifiers^30^. *MLL5* overexpression promoted cell proliferation and improved human engraftment along with GFP expansion. Yet, when we analyzed the amount of APL blast cells (defined by CD117^+^CD33^+^) *versus* myeloid committed cells (CD117^-^CD33^+^CD11b^+^) in the GFP^+^ fraction, we observed a ratio (APL blasts: myeloid committed) of 1:2 in MLL5 transplanted mice compared to 3:1 in control mice. Given, that the interplay between MLL5 and ATRA facilitates differentiation in vitro, it is conceivable that MLL5 drives differentiation also in vivo due to, the long-term exposure (12 weeks) of high levels of hematopoietic cytokines, such as IL3 and GM-CSF^31^. However, these differences could be abrogated over time. Whether the altered expression of *MLL5* gene is cause or consequence of the malignant myeloid transformation process remains unclear. Nevertheless, in the context of ATRA based therapy, our data suggests that the categorization of APL patients based on *MLL5* expression at diagnosis can have important prognostic values.

Our findings may be of particular interest if we consider epigenetic enzymes as *bona fide* targets for anti-AML drug development^32^. In this context, Schenk et al. demonstrated that inhibition of the LSD1 demethylase may reactivate the ATRA-differentiation pathway in non-APL AML^33^. Although the authors did not investigate the interaction between LSD1 and MLL5, it is conceivable that MLL5 mediates or potentiates the inhibition of LSD1 in the presence of ATRA, and in part, modulates the epigenome to improve drug response. One may argue that such an approach may be irrelevant for APL in light of the well-succeed state-of-the-art combination of ATRA and ATO^34–36^. That was one of the reasons why we decided not to evaluate APL overall survival in vivo in the context of MLL5. Yet, we support the belief of transferring the experience gained from a well-characterized disease, such as APL, to another more challenging leukemia subset. As a result, likewise to our APL study, our group observed increased responsiveness to ATRA induced differentiation when AML cell lines were transduced with MLL5 in vitro, which also led to significant tumor burden reduction in vivo (Almeida et al., 2018). Our data indicates a relevant crosstalk between the ATRA-induced differentiation pathway and histone H3K4 methylation, which suggests that epigenetic enzymes may constitute a promising therapeutic target for both APL and non-APL AML.

## Supplementary information

Supplemental material

Supplemental Figure 1

Supplemental Figure 2

Supplemental Figure 3

Supplemental Figure 4

Supplemental Figure 5

Supplemental table 3
