# Supplementary material for: MLL5 improves ATRA driven differentiation and promotes xenotransplant engraftment in acute promyelocytic leukemia model
Source: Cell Death Dis. 2021 Apr 6;12(4):371. doi: 10.1038/s41419-021-03604-z (PMC8024355; doi:10.1038/s41419-021-03604-z)
Supplement: Supplementary file 1 — Supplemental material [file 41419_2021_3604_MOESM1_ESM.doc]

***MLL5* improves ATRA driven differentiation and promotes xenotransplant engraftment in acute promyelocytic leukemia model**

Diego A Pereira-Martins^1,2,3,4ǂ^, Isabel Weinhäuser^1,2,3ǂ^, Juan Luiz Coelho-Silva^2,4,5^, Pedro L França-Neto^4^, Luciana Y Almeida^2^, Thiago M Bianco^2,5^, Cleide L Silva^2^, Rafael F França^6^; Fabiola Traina^2,5^, Eduardo M Rego^2,3^*, Jan Jacob Schuringa^1^*, Antonio R Lucena-Araujo^4^*

**Affiliations**: ^1^Department of Hematology, Cancer Research Centre Groningen, University Medical Centre Groningen, University of Groningen, Groningen, the Netherlands; ^2^Center for Cell-Based Therapy, University of Sao Paulo, Ribeirao Preto, Brazil; ^3^Hematology Division, LIM31, Faculdade de Medicina, University of Sao Paulo, Sao Paulo, Brazil; ^4^Department of Genetics, Federal University of Pernambuco, Recife, Brazil; ^5^Department of Medical Images, Hematology, and Clinical Oncology, University of Sao Paulo at Ribeirao Preto Medical School, Ribeirao Preto, Sao Paulo, Brazil; ^6^Department of Virology, Aggeu Magalhaes Institute/Oswaldo Cruz Foundation, Recife, Brazil.

^ǂ^D.A.P-M and I.W. contributed equally to this work.

*E.M.R, J.J.S and A.R.L-A. agreed to share the supervision of the study.

## Supplemental data

Results

## Supplemental figure 1A-B shows that cells infected with pMEG-MLL5 and shScrambled lentivirus efficiently overexpressed and downregulated the MLL5 protein. Supplemental figure 1C-D displays relative gene expression of MLL5 after overexpression and downregulation of the gene. Data was plotted with MLL5 expression from APL patients, for better comparability between the different models.

| 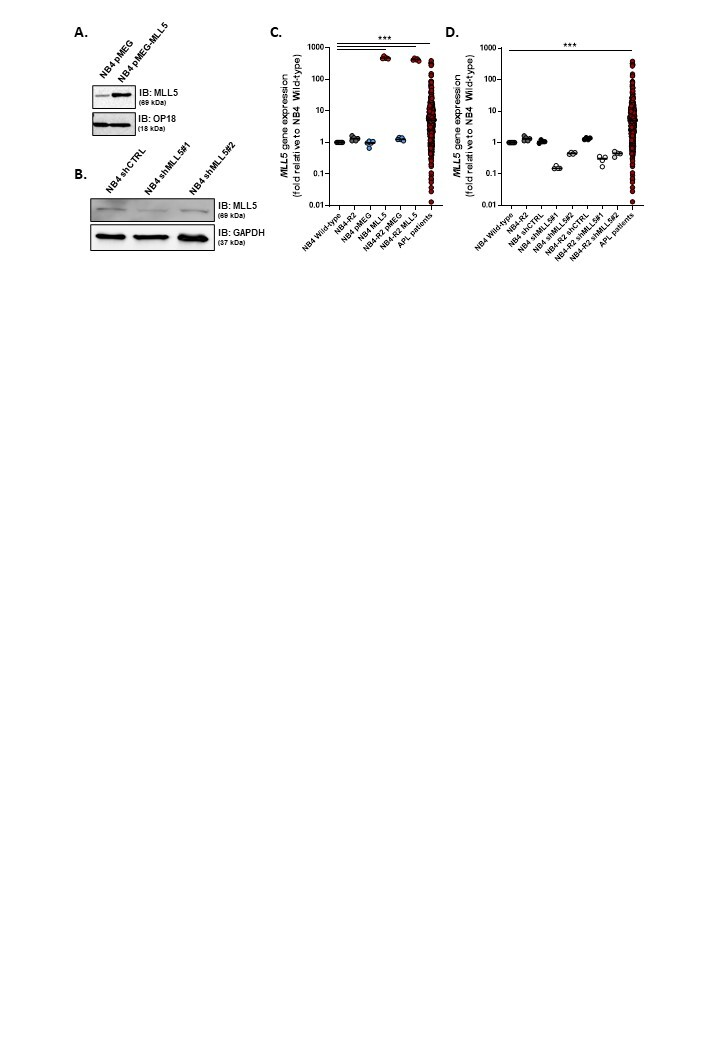 |
| --- |
| Supplemental figure 1. Expression of *MLL5* in APL cell lines upon lentivirally mediated overexpression or knockdown. (A) NB4 and NB4-R2 cells were transduced with lentivirus containing the empty vector (pMEG) or the *MLL5α* cDNA (pMEG-MLL5). In parallel, (B) APL cell lines were transduced with shScrambled (control) and two independent shMLL5 sequences (shMLL5#1 - TRCN0000154711 and shMLL5#2 - TRCN0000358557). Qualitative protein expression (western blot) and quantitative gene expressions of *MLL5* were analysed in pMEG-MLL5/shMLL5 cells relative to the pMEG/shScrambled cells, respectively. (C-D) Values were normalized to the expression level of housekeeping genes (*ACTB* and *GAPDH*). Results are shown as median values of, at least, 4 independent experiments. Data from both experiments (overexpression and knockdown) was normalized using the expression of wild type NB4 cells (internal control). Values in the graph represent a fold relative to internal control. Additionally, the last column for each graph (C-D) represents the MLL5 expression in APL patients, for comparison purposes. Each independent experiment was performed in triplicate. The Kruskal-Wallis with Dunn`s post-hoc test was used for statistical analysis. |

NB4 and NB4-R2 cells were transduced with two separate vectors for shMLL5 (shMLL5#1 - TRCN0000154711 and shMLL5#2 - TRCN0000358557; Sigma-Aldrich) and a shRNA sequence that does not target human genes (referred to as scrambled) was used as a control. Results were presented individually for the two shRNA vectors (herein called shMLL5#1 and shMLL5#2). Supplemental figure 2 displays the results of the MTT assay performed for NB4 and NB4-R2 cells transduced with MLL5 (A) and the results of NB4 an NB4-R2 MLL5-silenced cell proliferation curves (B), MTT assay (C), Ki-67 staining (D) and colony formation assays (E).

| 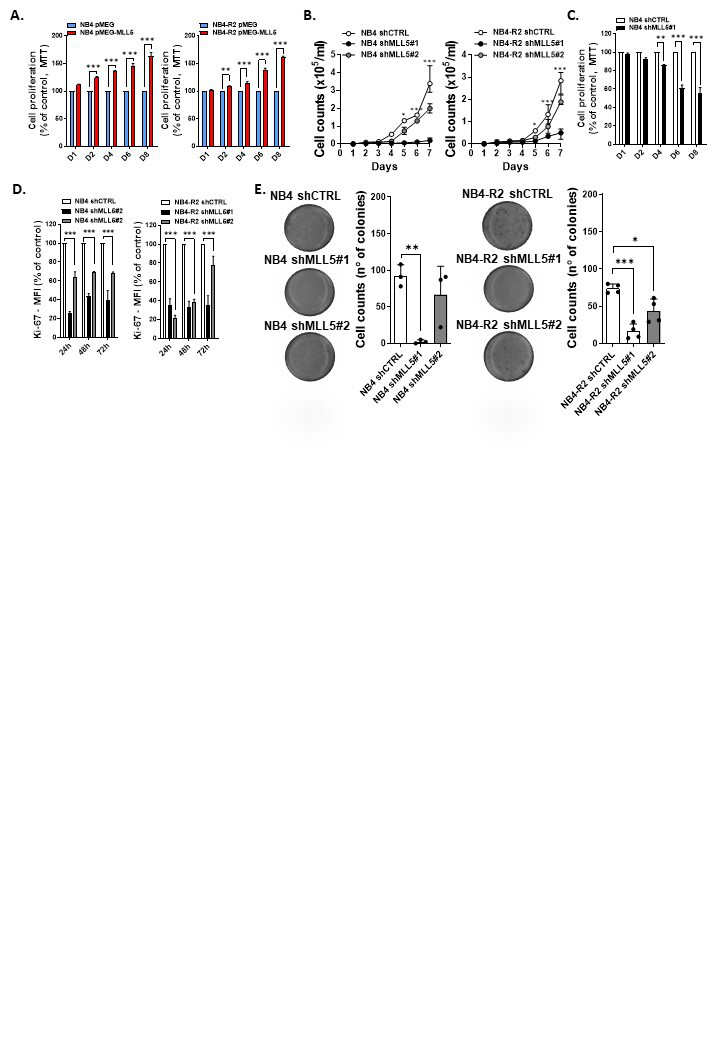 |
| --- |
| **Supplemental figure 2. Proliferation assays of *MLL5* knockdown cells**. (A) Cell viability was determined by methylthiazole tetrazolium (MTT) assays for NB4 and NB4-R2 cells transduced with MLL5 and empty vector control at day 1, 2, 4, 6 and 8. Bar graphs represent the mean ± SD of at least four independent experiments; An ANOVA test and a Bonferroni post-test was applied to calculate statistical significance; ***p < 0.0001 indicates the statistical significance of MLL5-transduced cells vs. empty vector control (pMEG). (B) Growth curves and (C) Ki67 staining analysis and (C) MTT cell viability assay of NB4 and NB4-R2 cell lines lentivirally transduced with shMLL5 or shScrambled (shCTRL, control). (D) Representative example of one of four independent experiments of colony formation assay in methylcellulose using lentivirally transduced NB4 and NB4-R2 cell lines. Graphic bars represent the number of colony-forming cells in each well. Data were expressed as mean ± standard error of the mean. * indicates *P* < 0.05. *** indicates *P* < 0.001. |

Supplemental figure 3A and 3B shows the number of apoptotic cells after *in vitro* treatment with ATO, ATO+ATRA or Ara-C at three time-points. After knockdown of *MLL5*, no difference on mtROS levels was observed (Supplemental figure 3C).

| 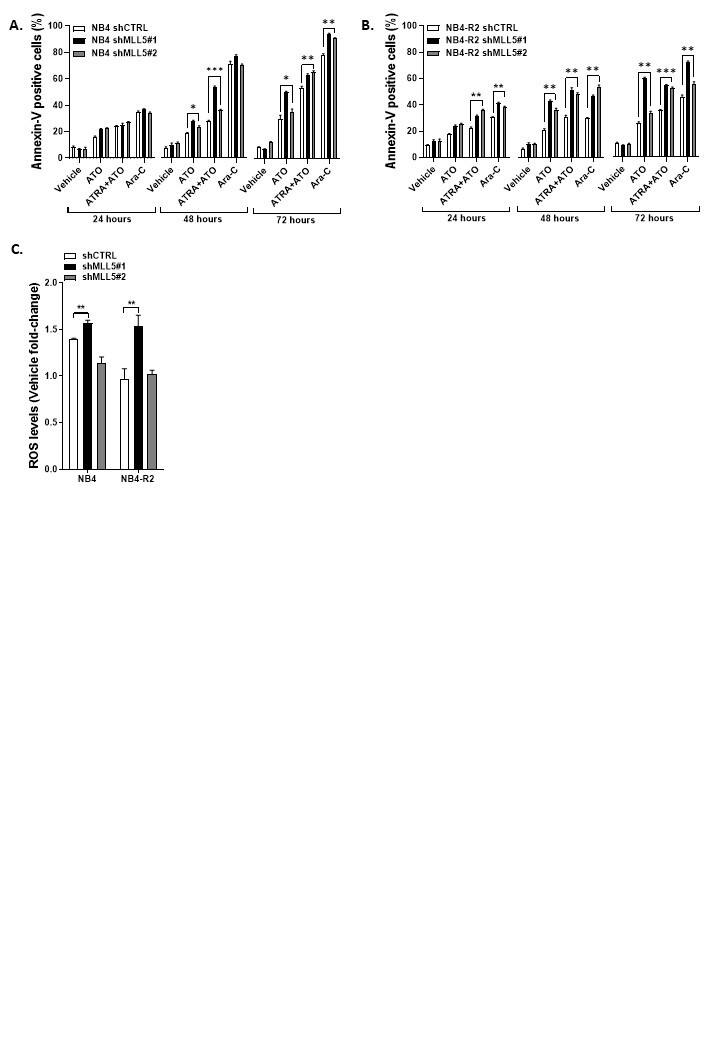 |
| --- |
| **Supplemental figure 3. Drug-induced apoptosis assay on MLL5 knockdown cells**. Percentage of apoptotic cells (A, NB4) and (B, NB4-R2) after 24, 48 and 72 hours in culture with apoptotic stimulus (ATRA 1µM; ATO µM and AraC 10 nM). C) Reactive oxygen species (ROS) in lentivirally transduced cell lines. NB4 and NB4-R2 after 12 hours of treatment with ATO 1 µM. All experiments were performed in triplicate. Data were expressed as mean ± standard error of the mean. * indicates *P* < 0.05. *** indicates *P* < 0.001. |

## Supplemental figure 4 shows how MLL5 overexpression impacts on granulocytic differentiation in NB4 and NB4-R2 cell lines infected with empty vector or pMEG-MLL5 lentiviruses.

| 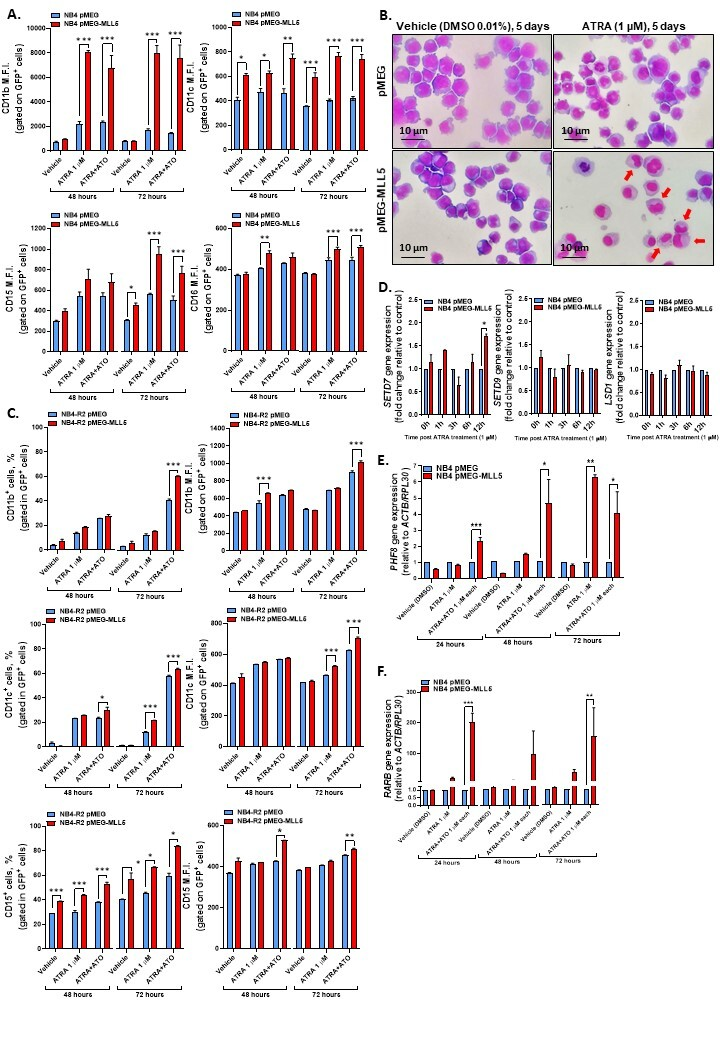 |
| --- |
| Supplemental figure 4. MLL5 overexpression results in increased granulocytic differentiation upon ATRA treatment, via PHF8/RARB activation. (A) Mean fluorescence intensity (MFI) of CD11b^+^, CD11c^+^, CD15^+^ and CD16^+^ in NB4 cells infected with empty vector or pMEG-MLL5 lentiviruses after 48 (left panel) and 72 (right panel) hours of ATRA (1 μM) alone or in combination with ATO (1 µM each) treatment used as the standard stimulus for differentiation. (B) Cell morphology of NB4 cells (pMEG, and pMEG-MLL5) analyzed by May-Grünwald-Giemsa staining after treatment for 5 days with ATRA (1 µM) and DMSO control (vehicle, 0.01%). Red arrows point to the signs of nuclear lobulation, suggestive of granulocytic maturation. (C) Percentage of cells (left panels) and mean fluorescent intensity (right panels) of CD11b^+^, CD11c^+^ and CD15^+^ in NB4-R2 cells infected with empty vector or pMEG-MLL5 lentiviruses after 48 (left panel) and 72 (right panel) hours of ATRA (1 μM) alone or in combination with ATO (1 µM each) treatment. (D) Mean fluorescence intensity of CD11b^+^ for NB4 cells (transduced with shCTRL, shMLL5#1 and shMLL5#2) after 72 hours of ATRA (1 µM) treatment as the standard stimulus for differentiation. Graphic bars represent the percentage of MFI in shMLL5#1 and shMLL5#2 cells, relative to IgG control. (E) Expression of SETD7, SETD9 and LSD1 in NB4-MLL5 expressing cells after 0, 1, 3, 6 and 12 hours of ATRA treatment (1 µM). (F) Expression of PHF8 and its downstream target RARB in NB4-MLL5 expressing cells after 24, 48 and 72 hours of ATRA (1 μM) alone or in combination with ATO (1 µM each) treatment. The relative expression was quantified by Real-time quantitative PCR (RQ-PCR) using GAPDH and ACTB as endogenous control. Data from continuous variable were all expressed as mean ± standard error of the mean. * indicates P < 0.05. *** indicates P < 0.001. NS indicates not significant. |

## Supplemental figure 5A shows the differential evaluation of GFP positive and negative subclones inside the human CD45^+^ cells in the bone marrow of MLL5-transplanted and control mice. Haematological parameters evaluated during the transplant (B) and absolute cell counts for GFP^+^ and CD11b^+^ cells can be found in Supplemental figure 5C. The levels of human MLL5 gene expression in the bone marrow of the sacrificed animals (week 12), were described in the Supplemental table 2.

| 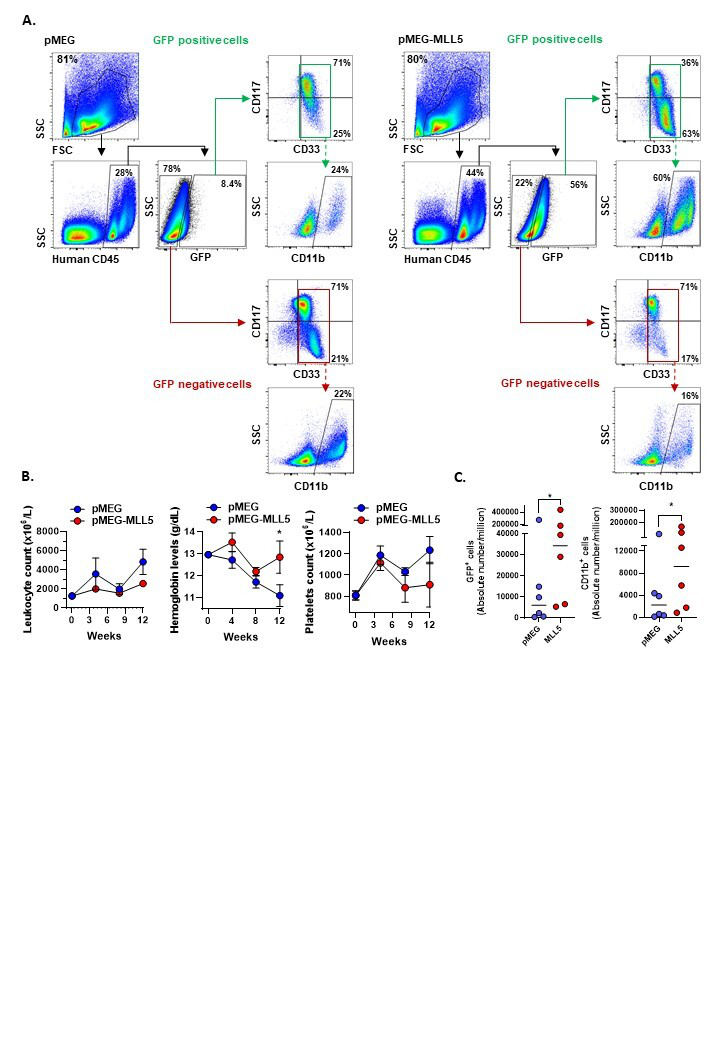 |
| --- |
| Supplemental figure 5. Immunophenotypic characterization of GFP positive and negative APL cells. (A) Representative FACS phenotype from a primary murine bone marrow transplanted with human APL blasts transduced with the empty vector (pMEG) or the MLL5 gene at the time of sacrifice. Different cellular populations were analysed inside GFP positive and negative population in the same sample (pMEG/MLL5). APL blasts and mature myeloid committed cells were analysed by flow cytometry using markers against CD117, CD33 and CD11b. (B) Dispersion graphs showing the leukocyte count, haemoglobin levels and platelet counts. (C) Scatter plots showing the absolute number of engrafted human CD45^+^GFP^+^ cells and CD45^+^GFP^+^CD11b^+^ cells in the murine bone marrow at sacrifice. Data were expressed as mean ± standard error of the mean. * indicates P < 0.05. *** indicates P < 0.001. |

| Supplemental table 1. Sources and properties of antibodies used in the work. | | | | | | | | | | |
| --- | --- | --- | --- | --- | --- | --- | --- | --- | --- | --- |
|  | **Antibody** | **Company** | **Catalog**  **Number** | | **Source** | **P/M**  **Clone** | **Mass**  **(kDa)/**  **Fluorochrome** | **Application** | **Dilution** | |
| **1** | MLL5 | Sta Cruz Biotechnology | | sc-377182 | Mouse | M | 69 | WB | | 1:1000 |
| **2** | CDKN1A | Sta Cruz Biotechnology | | sc-71811 | Mouse | M | 21 | WB | | 1:500 |
| **3** | H3K4me3 | Cell signaling | | #9727 | Rabbit | P | 17 | WB | | 1:1000 |
| **4** | Fibrillarin | Cell signaling | | #2639 | Rabbit | M | 37 | WB | | 1:1000 |
| **5** | OP18 | Sta Cruz Biotechnology | | sc-55531 | Mouse | M | 18 | WB | | 1:1000 |
| **6** | α-tubulin | Sta Cruz Biotechnology | | sc-5286 | Mouse | M | 53 | WB | | 1:10000 |
| **7** | GAPDH | Cell signaling | | #2118 | Rabbit | M | 37 | WB | | 1:10000 |
| **8** | Anti-Human CD45 | BD biosciences | | 557748 | Mouse | HI30 | PE-Cy7 | FC | | 1:100 |
| **9** | Anti-Human CD117 | BD biosciences | | 550412 | Mouse | YB5.B8 | APC | FC | | 1:100 |
| **10** | Anti-Human CD33 | BD biosciences | | 555450 | Mouse | WM-53 | PE | FC | | 1:100 |
| **11** | Anti-Human CD11b | BD biosciences | | 562632 | Mouse | IRCF44 | BV421 | FC | | 1:100 |
| **12** | Anti-Human CD11b | BD biosciences | | 557321 | Mouse | IRCF44 | PE | FC | | 1:100 |
| **13** | Anti-Human CD19 | BD biosciences | | 561295 | Mouse | HIB19 | PerCP-Cy5.5 | FC | | 1:100 |
| **14** | Anti-Human CD34 | BD biosciences | | 348057 | Mouse | 8G12 | PE | FC | | 1:100 |
| **15** | Anti-Human HLA-DR | BD biosciences | | 555561 | Mouse | TU36 | PE | FC | | 1:100 |
| **16** | Anti-Human CD38 | BD biosciences | | 555462 | Mouse | HIT2 | APC | FC | | 1:100 |
| **17** | Anti-Human Ki-67 | BioLegend | | 350504 | Mouse | SolA15 | PE | FC | | 1:100 |
| **18** | 7-AAD viability staining solution | BioLegend | | 420404 | - | - | - | FC | | 1:100 |
| **19** | Propidium Iodide solution | BioLegend | | 421301 | - | - | - | FC | | 1:100 |
| **20** | Annexin V | BD biosciences | | 550474 | - | - | APC | FC | | 1:100 |
| **21** | IgG Isotype Ctrl | BD biosciences | | 550474 | Goat | Poly24030 | PE | FC | | 1:100 |
| **Abbreviations: P, polyclonal; M, monoclonal; WB, western blot; FC, flow cytometry.** | | | | | | | | | | |

| **Supplemental table 2. Characteristics of APL patients’ samples used in this study for the xenograft in vivo model.** | | | | | | | | |
| --- | --- | --- | --- | --- | --- | --- | --- | --- |
| **Sample #** | **FLT3 status** | **NPM1 status** | **Source** | **Karyotype** | **Age at diagnosis** | **BCR isoform** | **Cq MLL5***  **(pMEG/MLL5)** | **Cq ACTB***  **(pMEG/MLL5)** |
| **1** | wt | wt | BM | t(15;17) PML-RARα | 35 | Bcr3 | N.D./30.98 | 18.11/18.74 |
| **2** | wt | wt | BM | t(15;17) PML-RARα | 48 | Bcr1 | N.D./36.45 | 20.05/17.72 |
| **3** | wt | wt | BM | t(15;17) PML-RARα | 27 | Bcr3 | N.D./35.99 | 22.97/21.72 |
| **4** | wt | wt | BM | t(15;17) PML-RARα | 34 | Bcr3 | N.D./34.48 | 16.58/18.23 |
| **5** | wt | wt | BM | t(15;17) PML-RARα | 29 | Bcr1 | N.D./28.58 | 22.28/23.01 |
| **6** | wt | wt | BM | t(15;17) PML-RARα | 37 | Bcr3 | N.D./35.42 | 16.66/18.72 |
| **Abbreviations: #, sample number; *FLT3*, fms-related tyrosine kinase 3; *NPM1*, nucleophosmin 1; wt, wild type; BM, bone marrow; BCR, break-point chromosomal region; Cq, cycle quantification; N.D. Not detected in three technical replicates. *Average values of three technical replicates.** | | | | | | | | |

**References**

1 Kitamura K, Kiyoi H, Yoshida H, Saito H, Ohno R, Naoe T. Mutant AF-2 domain of PML-RARalpha in retinoic acid-resistant NB4 cells: differentiation induced by RA is triggered directly through PML-RARalpha and its down-regulation in acute promyelocytic leukemia*. Leukem*ia 1997**;** 11: 1950–6.
