# Supplementary figures and images for: MLL5 improves ATRA driven differentiation and promotes xenotransplant engraftment in acute promyelocytic leukemia model
Source: Cell Death Dis. 2021 Apr 6;12(4):371. doi: 10.1038/s41419-021-03604-z (PMC8024355; doi:10.1038/s41419-021-03604-z)

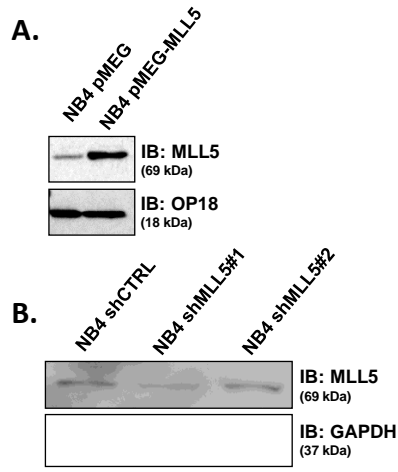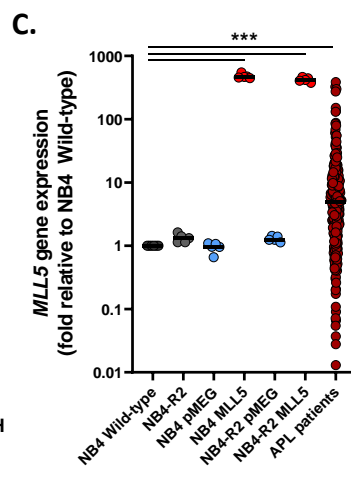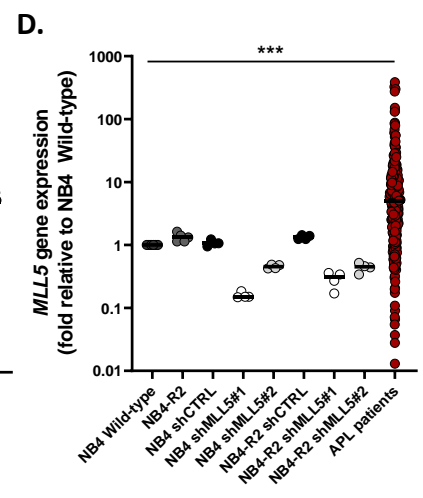

Supplement: Supplementary file 2 — Supplemental Figure 1 [file 41419_2021_3604_MOESM2_ESM.pdf]

**A.**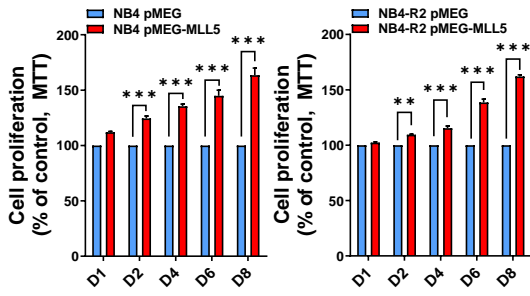**B.**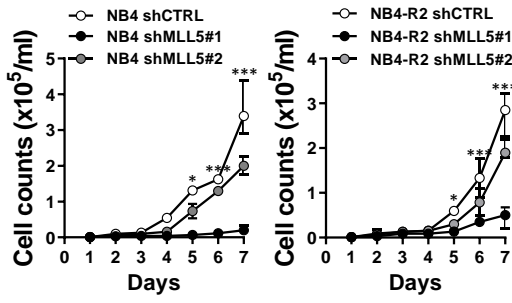**C.**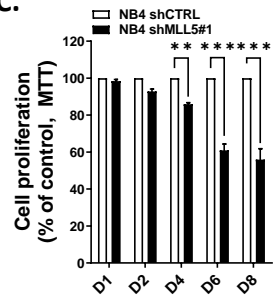**D.**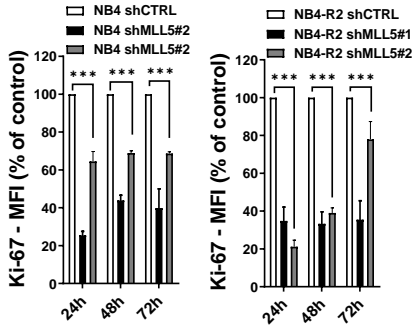**E.**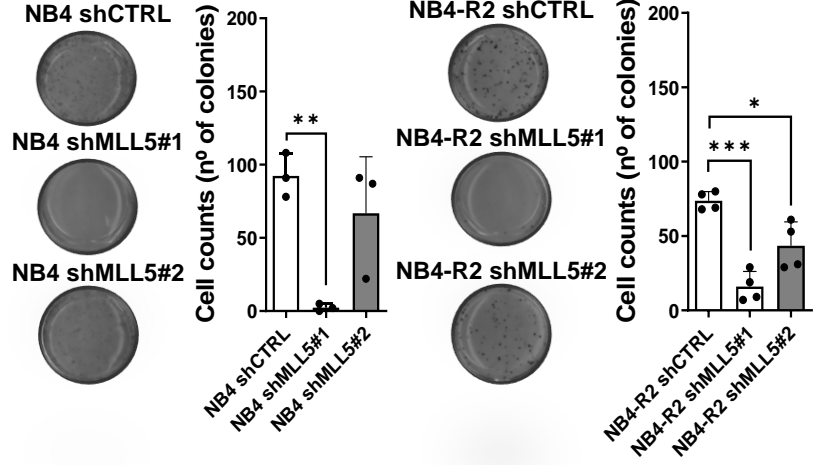

Supplement: Supplementary file 3 — Supplemental Figure 2 [file 41419_2021_3604_MOESM3_ESM.pdf]

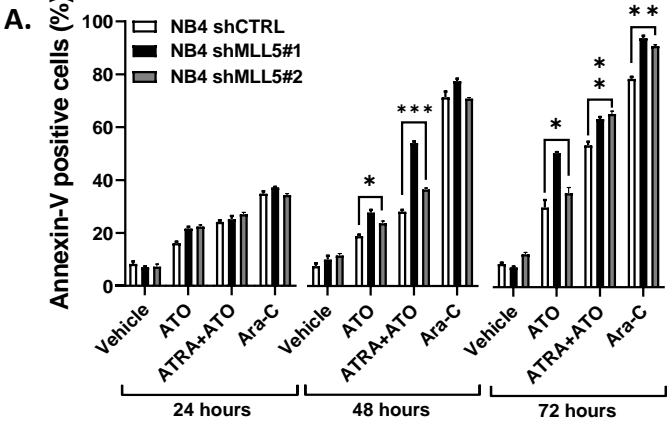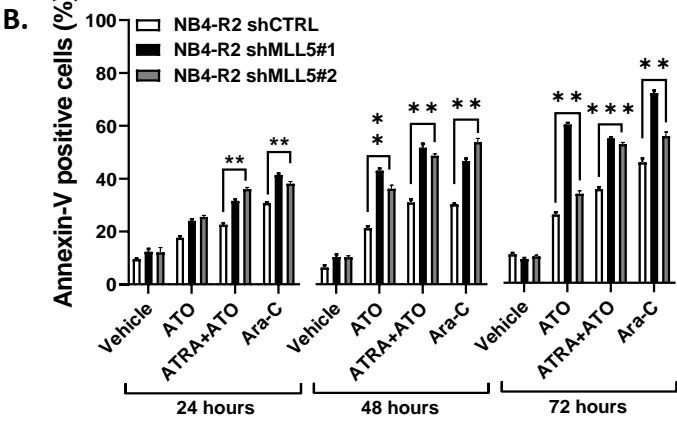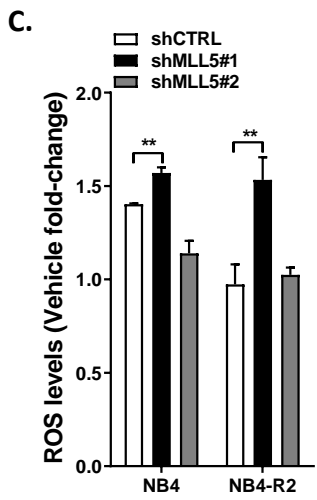

Supplement: Supplementary file 4 — Supplemental Figure 3 [file 41419_2021_3604_MOESM4_ESM.pdf]

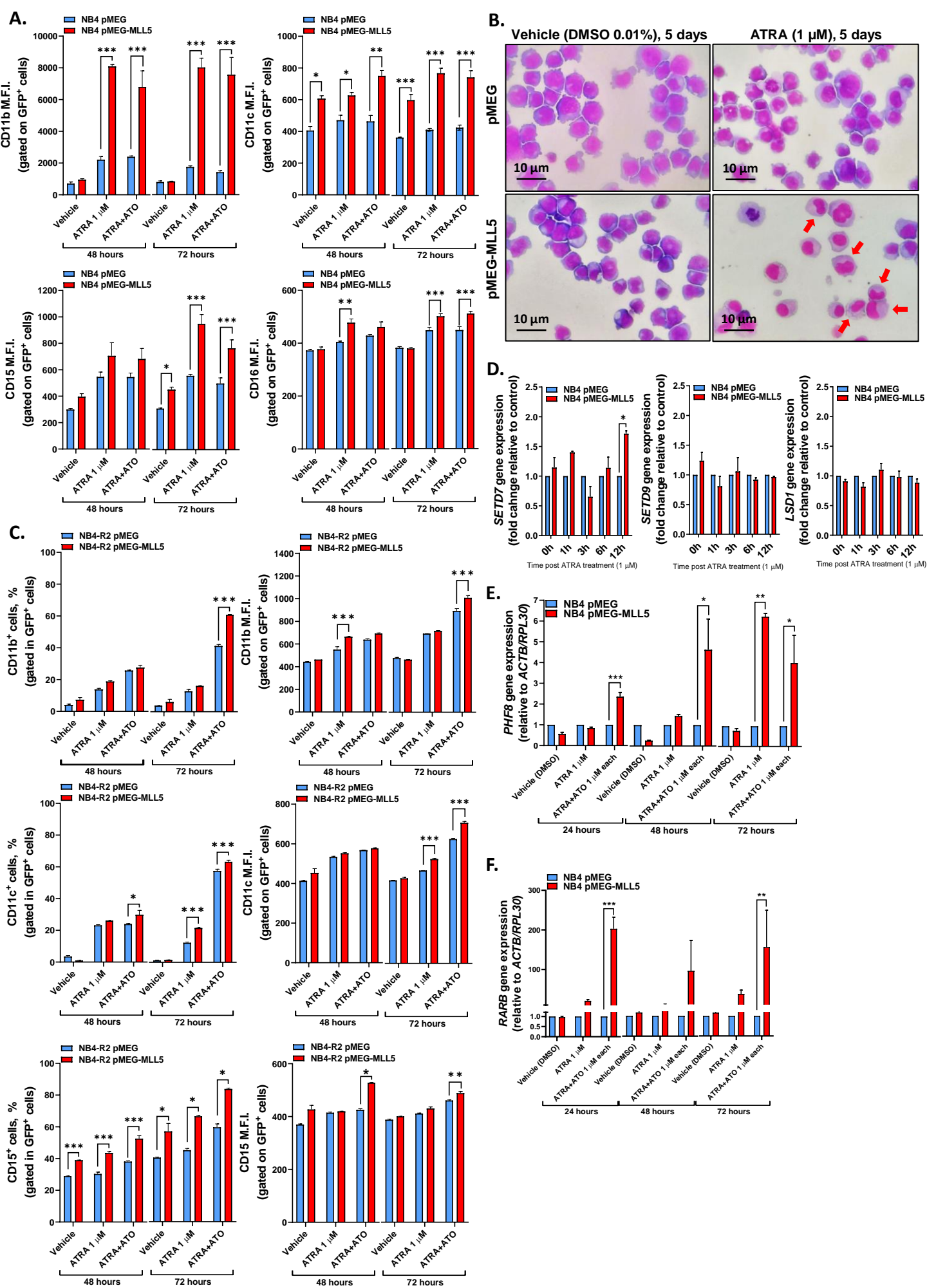

Supplement: Supplementary file 5 — Supplemental Figure 4 [file 41419_2021_3604_MOESM5_ESM.pdf]

**A.**

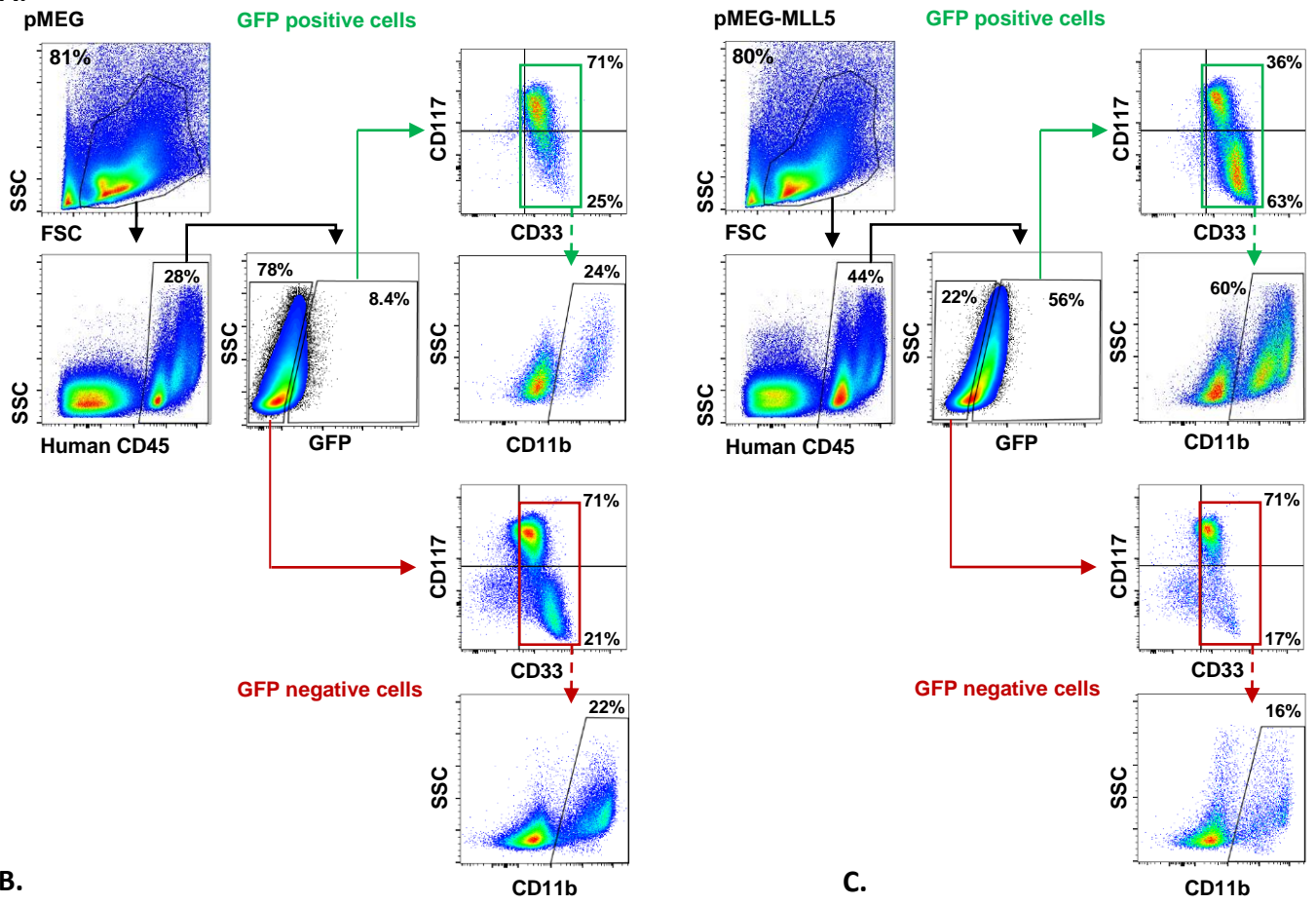

**B.**

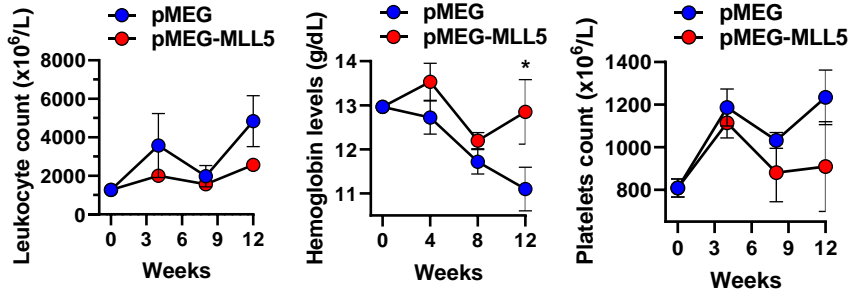

**C.**

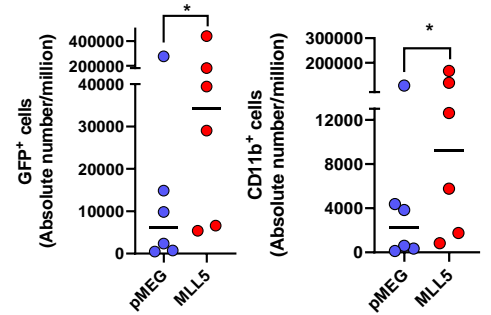

Supplement: Supplementary file 6 — Supplemental Figure 5 [file 41419_2021_3604_MOESM6_ESM.pdf]
